# Therapeutic Ultrasound as a Treatment Modality for Physiological and Pathological Ageing Including Alzheimer’s Disease

**DOI:** 10.3390/pharmaceutics13071002

**Published:** 2021-07-01

**Authors:** Jürgen Götz, Gina Richter-Stretton, Esteban Cruz

**Affiliations:** Clem Jones Centre for Ageing Dementia Research, Queensland Brain Institute, The University of Queensland, Brisbane, QLD 4072, Australia; g.richterstretton@uq.edu.au (G.R.-S.); e.cruz@uq.edu.au (E.C.)

**Keywords:** Alzheimer’s disease, aducanumab, amyloid, cognition, focused ultrasound, memory, neuromodulation, scanning ultrasound, tau, therapeutic antibodies

## Abstract

Physiological and pathological ageing (as exemplified by Alzheimer’s disease, AD) are characterized by a progressive decline that also includes cognition. How this decline can be slowed or even reversed is a critical question. Here, we discuss therapeutic ultrasound as a novel modality to achieve this goal. In our studies, we explored three fundamental strategies, (i) scanning ultrasound on its own (SUS^only^), (ii) therapeutic ultrasound in concert with intravenously injected microbubbles (which transiently opens the blood–brain barrier, SUS^+MB^), and (iii) SUS^+MB^ in combination with therapeutic antibodies (SUS^+MB+mAb^). These studies show SUS^+MB^ effectively clears amyloid and restores memory in amyloid-depositing mice and partially clears Tau and ameliorates memory impairments in Tau transgenic mice, with additional improvements found in combination trials (SUS^+MB+mAb^). Interestingly, both SUS^only^ and SUS^+MB^ restored the induction of long-term potentiation (LTP, electrophysiological correlate of memory) in senescent wild-type mice. Both lead to increased neurogenesis, and SUS^only^, in particular, resulted in improved spatial memory. We discuss these findings side-by-side with our findings obtained in AD mouse models. We conclude that therapeutic ultrasound is a non-invasive, pleiotropic modality that may present a treatment option not only for AD but also for enhancing cognition in physiological ageing.

## 1. Introduction

This review article is written from the perspective of entering, more than 25 years ago, the Alzheimer’s disease (AD) research space. At the time, in a collaborative effort, we had developed the ALZ7 mouse model for the Tau pathology of Alzheimer’s disease [1]. What we succeeded to model in the mice was a rather subtle cellular Tau pathology, and since then, many more advanced models have been generated, both by us and others, yet as we are writing these lines, it is still not fully understood which form of Tau is the major toxic form and, hence, which Tau species needs to be targeted for therapeutic intervention [2,3].

After contributing over many years to an understanding of how Tau and amyloid-β (Aβ), the other major player in AD, interact and cause AD, we made our first steps into the therapeutic ultrasound space in 2012. Rather than using a Tau transgenic mouse model, in our team’s first study, we treated the Aβ-depositing APP23 mouse with ultrasound in a scanning mode (SUS), which reduced pathology and restored cognition [4]. The restorative effects on cognition were unexpected yet encouraging. We had entered the therapeutic ultrasound space when this research field was already quite advanced [5]. Yet, despite the fact that the first clinical trials using low-intensity ultrasound have already been conducted [6,7], the field is still struggling with ways to apply therapeutic ultrasound effectively and uniformly to the entire brain, and it remains to be shown whether this technology can in fact restore cognition in AD patients. To what extent this outcome depends on the chosen sonication parameters is also not known. The collective work in animals and humans is encouraging, and the field is literally exploding [8,9,10]. Our team feels, by merging our core activities in basic AD research and therapeutic ultrasound development, that we can contribute in a meaningful way with the aim to develop ultrasound into a modality for treating diseases of the brain such as AD.

In the following, we will first provide a short overview of AD and what sets it broadly apart from physiological ageing. Appreciating the overlap between physiological and pathological ageing such as AD is relevant for developing therapeutic interventions. Very recently, we started to apply SUS to senescent wild-type mice, and our data indicate that this approach may be useful as a cognition enhancement tool [11]. We then discuss the current treatment options for AD, what the clinical trial landscape looks like, and which particular challenges are being encountered as one aims to develop a cure or even a disease-modifying therapy for AD. We will briefly mention the principles of therapeutic ultrasound and discuss what renders this technology fundamentally safe. This will lead to our preclinical work in wild-type and AD mice as well as larger animal species, hinting at safety and efficacy, as the field moves forward into clinical trials. The succeeding paragraph will address the ongoing clinical trials using therapeutic ultrasound and how the approaches fundamentally differ. The review will be concluded by discussing the inherent challenges in applying the ultrasound technology to AD and other brain diseases, finishing with a note of encouragement as we believe that there is a vast potential in this technology as it is non-invasive and allows for either localized treatments (by targeting a small area such as the substantia nigra in Parkinson’s disease) or global treatments (by targeting the entire brain as may be required for AD).

## 2. Alzheimer’s Disease–A Disorder of Pathological Ageing

Age is the major risk factor for dementias and neurological diseases more generally. The incidence of AD as a major dementia is doubling every five years after the age of 65, and currently, there are around 32 million people with AD worldwide, at a cost of over US$1.1 trillion, with total cases expected to rise to 100 million by 2050 (Alzheimer’s Association 2018). Globally, 9 million people died from neurological disorders in 2016 [12]. This corresponds to a loss of 276 million disability-adjusted life-years. Neurological disorders account for close to 17% of all deaths globally, second only to cardiovascular disease. AD and other dementias account for a quarter of these deaths.

Prevalent dementias other than AD are frontotemporal lobar degeneration (FTLD), dementia with Lewy bodies, and vascular dementia. Dementia is also present in movement disorders such as Parkinson’s disease (PD). What these diseases have in common is the accumulation of insoluble protein deposits in the brain as a result of either an increased production or impaired clearance. In fact, the assumption shared by many researchers is that these proteins and their aberrant aggregation are causal and drive disease progression. What are these proteins in AD? Alois Alzheimer and Oskar Fischer, more than a century ago, independently described the cardinal histopathological features of the disease that now bears the Alzheimer’s name [13]. With light microscopic examination of histologically stained brain sections, they captured the two most prominent hallmarks; extracellular amyloid plaques (which contain oligomeric and fibrillar forms of the aforementioned Aβ peptide) and intracellular neurofibrillary tangles (which contain the microtubule-associated protein Tau in its oligomeric and fibrillar forms). Pathological Aβ and Tau are thought to drive, at least in part, the loss of synapses and neurons in vulnerable brain regions, leading to the symptoms commonly associated with AD.

A causative role for Aβ in disease pathogenesis is further supported by observations in familial AD, which accounts for up to an estimated <3% of all cases. Here, autosomal dominant mutations have been identified in genes encoding the amyloid precursor protein, APP (from which Aβ is derived by proteolytic cleavage), and components of the protein machinery that generates Aβ (presenilin-1 and -2). While no causative mutations have been identified in the Tau-encoding *MAPT* gene, they have been identified in a significant subset of FTLD with Tau (FTLD-Tau). Importantly, in AD, Tau has been more closely linked to dementia than Aβ [14]. Together, this points to Aβ and Tau as pathogenic agents. The situation is less clear for the predominantly sporadic cases of AD and FTLD-Tau, which have a later age of onset. Because the two forms (sporadic and familial) do not differ in their general histopathological features and clinical presentation, it has been suggested that while the initiating, upstream signals might differ, they likely converge in a common downstream pathogenic signalling pathway.

In modelling AD in animals, transgenic mouse models have been particularly useful. The early mouse models targeted the expression of AD-relevant proteins to neurons in general, whereas region-specific and cell-type-specific approaches, as well as inducible systems, are increasingly being employed to understand, for example, aspects of regional vulnerability and the spreading of AD pathology. Such brain area and cellular specificity can be achieved by introducing the gene of interest under the control of specific promoters and regulatory elements [15]. In the animal modelling of AD, a major emphasis was initially placed on reproducing key lesions that are specific to the human disease. Despite the opportunities offered by these transgenic models, several caveats remain. The human transgenes contain either no or not all non-coding sequences, making it impossible to study human genomic interactions and the role of splice variants. Although these models were crucial in proving the role of distinct genes and their associated mutations in AD, the human pathology is generally only incompletely recapitulated. Compared with the Aβ plaques in human brains, those in many rodent models are either diffuse or, even when they are condensed, exhibit fewer crosslinked fibrils. Neurofibrillary tangles have proved to be even more difficult to model, as even when Tau filament-like structures form, they appear different from those in human brains on the basis of negative-stain electron microscopy. Another caveat, intrinsic to the way in which transgenic animals are usually generated, is that neither the integration site nor the copy number of the inserts can be controlled, leading to a large and uncontrollable range of expression patterns and levels, a situation that is further complicated by the potential for integration artefacts. Another concern regarding overexpression models is the presence of secondary effects. In an attempt to overcome what is seen by some as an overexpression artifact of the classical Tau transgenic models, knock-in mice have been generated, some of which are now more widely used [16,17]. Nevertheless, these models also have their limitations. As is the case with transgenic mice, they recapitulate only certain aspects of the disease. For example, APP knock-in mice (with or without additional mutations) do not present with Tau pathology. To generate Aβ deposits, these models also combine several gene mutations that do not co-occur in AD and that could complicate the analysis of downstream effects.

So far, our discussion has focused on Aβ and Tau; however, the field has produced many hypotheses to explain what initiates and drives the pathogenic process for the predominantly sporadic forms of AD. These range from impaired neurotransmitter systems as presented by the cholinergic hypothesis, a role for mitochondrial dysfunction (shared e.g., with Parkinson’s disease), inflammation including changes to the innate immune system, viral infections, and an interaction between the nervous system and the gastrointestinal tract, which consists of multiple connections, including the vagus nerve, the immune system, and bacterial metabolites and products [18]. However, even if one were to focus on Aβ and Tau only, the challenge remains, as these species undergo a process that generates different sizes (proteolytic cleavage in the case of Aβ and alternative splicing plus proteolytic cleavage for Tau), a host of posttranslational modifications (Tau undergoes acetylation, O-linked N-acetyl-glucosamination, ubiquitylation, sumoylation, methylation, glycosylation, isomerization, and truncation, whereas Aβ undergoes phosphorylation, glycosylation, and isomerization), and a process of oligomerisation and fibrillization that leads to extracellular amyloid plaques and intraneuronal Tau-containing neurofibrillary tangles [3].

Finally, it needs to be acknowledged that age is the most important risk factor for AD. Consumer organisations would define AD in an operational manner: Regarding short-term memory and learning new information, possible changes due to normal ageing may be sometimes forgetting people’s names or appointments but remembering them later, whereas in people with dementia, they may forget the names of close friends or family, or they forget recent events such as visitors they had that day. The question thus arises in which aspects healthy, physiological ageing can be discriminated from AD. A recent review discussed nine hallmarks of ageing: stem cell exhaustion, altered intercellular communication, genomic instability, telomerase attrition, epigenetic alterations, loss of proteostasis, deregulated nutrient sensing, cellular senescence, and mitochondrial dysfunction, and all these aspects are more severely affected in AD [19]. Structurally, healthy ageing is more strongly associated with a decline in frontal regions, whereas middle-aged individuals more likely to develop AD have shown greater grey matter reductions in dorsolateral and medial prefrontal, parietal, and lateral temporal regions. They have also shown a loss of white matter integrity in regions including the cingulum, corpus callosum, superior longitudinal fasciculus, and left uncinate fasciculus. Additionally, midlife volumetric reductions in the fronto-striatal executive network seem to be a normal part of ageing, whereas reductions in the medial temporo-parietal episodic memory network seem to indicate pathological ageing [20]. Finally, entorhinal cortex and hippocampal atrophy rates appear to diverge in healthy and pathological brain ageing, but it is not yet known whether this divergence is relevant to midlife [21]. Despite these differences, discriminating healthy brain ageing and changes of AD remains a grey zone [22]. Given the overlap between physiological and pathological ageing, one could argue that by slowing the ageing process or aspects thereof, neurodegenerative processes such as those in an AD brain could also be slowed down.

## 3. Alzheimer’s Disease Therapies–Treatment Options and Challenges

The current treatment of AD is largely only providing symptomatic relief. With the observation of a degeneration of (although not exclusively) cholinergic neurons, the foundation was laid for the development of acetylcholine esterase inhibitors (donepezil, rivastigmine, and galantamine) that stabilize the neurotransmitter acetylcholine in the synaptic cleft. These drugs are prescribed to patients with mild to moderate AD; however, they do not target the underlying degenerative process. The drugs were approved between 1996 and 2001 under the proprietary names Aricept, Exelon, and Razadyne. The only other drug that has been approved is memantine (in 2003 under the proprietary name Namenda), which is prescribed to cases of moderate to severe AD. This drug is an antagonist of the NMDA receptor, blocking excitotoxicity.

In the past decades, more than 200 clinical trials have been performed, resulting in zero approvals as AD drugs. In a recent assessment of the clinical trial landscape, with a censor date of 27 February 2020, 121 agents were in clinical trials for the treatment of AD. Twenty-nine of these were in 36 phase III trials, 65 agents in 73 phase II trials, and 27 agents in 27 phase I trials. Twelve agents in trials targeted cognitive enhancement, and 12 were intended to treat neuropsychiatric and behavioural symptoms. There were 97 agents in disease modification trials. Interestingly, compared to the 2019 pipeline, there was an increase in the number of disease-modifying agents targeting pathways other than Aβ or Tau [23].

Of the strategies targeting Aβ and Tau, comprehensive data are available for immunotherapies, a strategy that was first adopted for Aβ and then Tau. Following disappointments in the wake of failures of a whole battery of therapeutic antibodies in meeting their primary end points, the outlook is now more positive following the recent U.S. Food and Drug Administration (FDA) approval of aducanumab, an anti-Aβ antibody that targets Aβ aggregates including insoluble fibrils and soluble oligomers by binding to the amino terminus of Aβ at residues 3–7 in a shallow pocket in the antibody [24]. This human IgG1 antibody had been isolated from the B cells of cognitively healthy elderly humans and has low affinity for monomeric Aβ [25]. Of four clinical-stage anti-Aβ antibodies studied (aducanumab, gantenerumab, bapineuzumab, and solanezumab), only aducanumab had an impact on the aggregation kinetics and production of oligomeric aggregates [26].

In a Biogen-sponsored phase Ib clinical trial (PRIME) of aducanumab in prodromal and mild AD patients, a striking reduction in amyloid plaques, as measured by positron emission tomography, was reported following one year of monthly intravenous antibody infusions at doses ranging from 3–10 mg/kg. One of the two phase III trials of aducanumab, EMERGE, unlike ENGAGE, showed reductions in cognitive decline, possibly reflecting the effects of higher accumulated doses of the antibody [27]. Biogen was granted conditional FDA approval for aducanumab on 7 June 2021, pending a post-market commitment of a phase IIIB re-dosing trial that has recently been launched, becoming the first anti-amyloid agent and first antibody treatment for AD. However, it remains to be determined whether aducanumab is a disease-modifying therapy that achieves significant clinical benefits, i.e., improves cognition, in AD patients [28]. The interpretation of the cognitive data from these trials is complex because, during the trial, dosing was altered and stopped, and the magnitude of the cognitive effect was relatively small. Sevigny and coworkers demonstrated 50% plaque reduction in APP mutant Tg2576 mice after treatment with a mouse IgG2a aducanumab analogue; however, the effects of the immunotherapy on behavioural read-outs in mice have not been reported [25]. Antibodies in ongoing advanced clinical trials include Eli Lilly’s donanemab, which targets a specific form of Aβ (N3pG-Aβ); Biogen’s BAN2401 (which bears similarities with aducanumab); Eli Lilly’s solanezumab, which binds to monomeric Aβ; and Roche’s gantenerumab, which recognizes a conformational epitope. In this context, it is important to note that deposition of Aβ in the brain is not sufficient to develop dementia, and similarly, removing the peptide in patients with AD by itself is not sufficient to restore their cognitive functions [29].

Conceptually, targeting Tau in AD may present a more compelling approach than targeting Aβ, insofar as Tau burden correlates better with cognitive decline, while simultaneously posing the nontrivial challenge of targeting a protein that is primarily intraneuronal [14,30]. With limited exceptions, therapeutic monoclonal antibodies are of an IgG isotype, predominantly binding to pathogens in extracellular compartments. Anti-Tau IgGs on the other hand must penetrate both the blood–brain barrier (BBB) and the neuronal membrane to induce neutralization and/or clearance of intracellular pathogenic species of Tau. The Tau antibody approach could circumvent this issue if prion-like spreading does indeed play a central role in disease progression, as neutralizing Tau seeds in the extracellular compartment could in principle slow down spreading without the need for neuronal uptake [31]. On this ground, most of the earliest Tau passive immunotherapies in clinical trials were aimed at targeting extracellular Tau, and lead candidate screening is still often performed by assessing the antibody’s capacity to neutralize extracellular Tau seeds [32,33]. Yet, pathological Tau predominantly resides within neurons, and to what degree spreading effectively contributes to Tau pathology remains unclear. Hence, it is likely that targeting both intra- and extracellular pools of Tau will maximize efficacy. To this end, it follows that therapeutic ultrasound could be beneficial as a combination treatment by increasing the fraction of circulating antibody that reaches the brain parenchyma and targeted neurons.

Tau immunotherapy is still in its early days of development relative to the anti-Aβ field, with a total of ten antibodies currently in early clinical trials of AD and other primary tauopathies, six of which have advanced to phase II trials. Perhaps unsurprisingly, two of the latter antibodies targeting extracellular Tau, Biogen’s gosuranemab and AbbVie’s tilavonemab, have both been discontinued as candidates for progressive supranuclear palsy (PSP) after their respective phase II trials failed to meet efficacy primary endpoints [34,35]. These outcomes are relevant to the argument at hand in that targeting extracellular Tau may not be sufficient to modify disease progression. This approach may be particularly ill-suited in PSP given that evidence for prion-like spreading is much more limited than in AD and that Tau levels in cerebrospinal fluid (CSF) are not as significantly increased [36,37]. Both antibodies continued into phase II trials in AD with the hope that targeting extracellular Tau will yield more promising outcomes in the AD pathological context. Additional disappointments were recently released at the AD/PD 2021 conference with preliminary results reported of the phase II TAURIEL trial (NCT03289143) showing that Roche’s semorinemab decreased CSF Tau, but not other markers of inflammation and degeneration (e.g., sTREM2, IL-6, or NfL) in patients suffering from mild AD.

More recent trends in Tau antibody development have shifted from predominantly targeting Tau’s N-terminus to targeting the protein’s mid-region, as well as phosphorylated epitopes characteristic of toxic species. Although several recent studies have demonstrated the clear superiority of mid-region antibodies in blocking proteopathic spread in vitro, it remains to be validated whether these preclinical studies accurately predict clinical performance [33,38]. Of the newer generation of Tau antibodies, only Janssen’s JNJ-6373657 and UCB’s UCB0107 have recently advanced to phase II trials, and Eisai’s E2184 was recently selected as the biological anti-Tau arm in the DIAN-TU trial. Phase II AD trials for Eli Lilly’s zagotenemab and tilavonemab are due for completion in 2021, and efficacy outcomes could be decisive in shaping the future of Tau immunotherapies.

Why is it so difficult to find a treatment for AD? Drug development in general is a challenging field: drugs must enter the bloodstream, reach their target organ, and then exert a therapeutic effect. Different forms of drug administration pose different levels of challenges. These issues are present in most forms of drug delivery, and the industry has developed ways to overcome them. However, for neurological disorders, there is an additional challenge that the pharmaceutical industry has to overcome: the BBB. Nearly all large drug molecules and 98% of small molecules cannot pass the BBB. In comparison to the one-layer epithelium in other organs, the BBB is formed by a tight neurovascular unit (NVU) of endothelial cells (ECs), pericytes, and astrocytes that interact to create a tight barrier. Furthermore, even if a drug were to enter past the tight junctions of ECs, the ECs and astrocytes are equipped with a large variety of efflux pumps, which recognize a wide range of molecules and export them back into the bloodstream. The extreme limitations on molecular structure and cut-off size imposed by the BBB create a trade-off that has been difficult to balance thus far. To design an effective drug, pharmaceutical companies first need to create a molecule that they hypothesize will have the desired effect on neurons when it enters the brain. Additionally, there are requirements for the drug to make it into the bloodstream, survive, and then access the brain. These properties are often contradictory to those needed to cross the BBB.

Additional challenges for AD and other dementias are the incomplete understanding of the etiology and progression of the disease (as discussed further up); a failure to diagnose at presymptomatic stages; a lack of robust and sensitive biomarkers (which presents a problem for validating clinical trials and showing efficacy); in some cases, a short therapeutic window; and the inaccessibility of damaged or degenerating brain tissue.

## 4. Principles of Therapeutic Ultrasound as a Tool to Safely and Transiently Open the Blood–Brain Barrier

Five principal barrier interfaces protect the adult brain: (i) The BBB is the most selective barrier [39], separating the lumen of cerebral blood vessels from the brain parenchyma, an effect facilitated by tight adherens junctions between endothelial ECs that restrict the permeability of the paracellular cleft, and a paucity of pinocytotic vesicles, thereby limiting transcytoplasmic transport. At a cellular level, the BBB is formed by the multicellular NVU, which includes ECs as well as surrounding pericytes, glia, and neurons, and an ensheathing basement membrane. (ii) The blood–CSF barrier (BCSFB) is located at the choroid plexus, a spongy, vascularised structure that produces CSF and is attached to the wall within the ventricles of the brain [40]. Unlike the ECs of the BBB, choroid cells contain ample pinocytotic vesicles and are therefore able to transport macromolecules, such as proteins, from the blood into the CSF [41]. (iii–v) Additional barriers are the arachnoid barrier, the circumventricular organs, and the ependyma.

Various modes of transport of physiological cargoes are employed at the BBB and BCSFB [39]. Gases such as oxygen and carbon dioxide readily diffuse across the BBB following a concentration gradient. In addition, a wide range of lipid-soluble molecules can enter the brain by diffusion, depending on their lipid solubility, molecular weight (<500 Da), and hydrogen bonding capacity (<10 hydrogen bonds). The cerebral endothelium also expresses specific solute transporters that facilitate carrier-mediated transport of, e.g., glucose, amino acids, nucleotides, and vitamins. In addition, selective peptides and even large proteins can enter the brain by receptor-mediated transcytosis. Efflux mechanisms also contribute to barrier function, with ATP-binding cassette (ABC) transporters actively pumping drugs and their conjugates, xenobiotics, endogenous metabolites, and nucleosides across the luminal side of the ECs into the blood circulation. The ABC transporter P-gp is involved in clearing Aβ that accumulates in AD brains. Together, these features present a formidable challenge for drug delivery into the brain. A good example is intravenously administered therapeutic antibodies of which only ~0.1% enter the brain [42]. The challenge therefore resides in increasing brain uptake of existing drugs, as well as achieving uptake and retention of therapeutics for those disease indications for which no brain-penetrating drugs are available. Here, therapeutic ultrasound presents a viable option.

Ultrasound is a mechanical pressure wave with frequencies above the range of human hearing (20 kHz). This modality has been widely used for diagnostic imaging, whereby a transducer transmits a sound wave that is partially reflected at tissue interfaces in the patient, with the echoes being detected by the same transducer. Therapeutic ultrasound generally uses a lower frequency than that used for imaging, with its effects also depending on the acoustic pressure applied. Focused ultrasound continuously applied at high pressures results in tissue heating and has applications based on stereotactic ablation [43]. Thermal effects of focused ultrasound have led to therapeutic applications in lithotripsy (destruction of gall and kidney stones), physiotherapy, and oncology (destruction of bone metastases, prostate cancer, and uterine fibroids) as well as emerging neurosurgical applications for tissue ablation in brain tumours. Ultrasound-mediated thermal ablation of thalamic brain structures for the treatment of essential tremor [44,45] and a range of psychiatric disorders, as discussed elsewhere [46,47].

At lower pressures, focused ultrasound utilizes microbubbles that localize and amplify the mechanical effects of the sound wave on the vasculature, producing biological effects due to transient BBB opening. Microbubbles are preformed and biologically inert, and they are routinely used today as ultrasound contrast agents. They contain a lipid or polymer shell about 1–10 μm in diameter encapsulating a dense, inert gas (generally perfluorocarbon) and are commercially available under trade names such as SonoVue and Definity. Circulating microbubbles oscillate when they pass through the ultrasound field, imparting mechanical forces to the surrounding vasculature in the focal zone. Low-intensity ultrasound causes sustained, low-amplitude oscillations (stable cavitation), which lower the cavitation threshold, enabling more than a 100-fold reduction of the acoustic power required to achieve BBB permeability. Because increased intensities can cause transient microbubble collapse (inertial cavitation), thereby damaging blood vessels and the surrounding tissue, this phenomenon needs to be avoided through careful monitoring. The road toward the seminal idea that focused ultrasound could mediate BBB opening was paved by Bakay and colleagues, who were the first to demonstrate increased BBB permeability in regions surrounding lesions [48]. Hynynen and colleagues then demonstrated that by intravenously injecting preformed, exogenous microbubbles, focused ultrasound can achieve BBB opening at a significantly lower acoustic power, thereby significantly reducing thermal effects and providing a safe, transient, and reproducible opening [49].

Although the precise mechanisms leading to ultrasound-mediated BBB opening remain underexplored, oscillating microbubbles are known to elicit specific cellular changes at the NVU. Ultrasound firstly causes a disruption of tight junctions. Then, it causes an increase in the number of vesicles, vacuoles, fenestrations, and transcellular channels [50]. In addition, ultrasound-mediated BBB opening in rats has also been associated with an upregulation of caveolin, a protein important for endocytosis [51], altogether suggesting an increase in transcytoplasmic transport. We found, using caveolin-1 knock-out mice, that caveolin-1 has a critical role specifically in the transport of large (500 kDa), but not smaller (3 and 70 kDa), cargoes [52]. In applying the technology to the human brain, the challenge arises that the human skull is highly attenuating in comparison to rodent skulls [53]. Of note, this does not pose a practical problem when experimenting with mice [54].

## 5. Therapeutic Improvements in Animal Models for Alzheimer’s Disease Using Therapeutic Ultrasound

In the following, we will be mainly focusing on work done in our lab. Our team has contributed to applying therapeutic ultrasound to models of pathological ageing (i.e., AD) as well as physiological ageing. We have developed a scanning ultrasound (SUS) approach by which the ultrasound is moved in a zigzag mode over the entire skull of a mouse. In most of our studies, we have been using the Therapy Imaging Probe System, (TIPS, Philips Research) in combination with either in-house or commercially available microbubbles (Definity). The TIPS system consists of an annular array transducer with a natural focus of 80 mm, a radius of curvature of 80 mm, a spherical shell of 80 mm with a central opening of 31 mm diameter, a 3D positioning system, and a programmable motorized system to move the ultrasound focus in the x and y planes to cover the entire brain area [55]. A coupler mounted to the transducer was filled with degassed water and placed on the head of the mouse with ultrasound gel for coupling to ensure unobstructed propagation of the ultrasound to the brain. Standard parameters for the ultrasound delivery were 1 MHz centre frequency, 0.7 MPa peak rarefactional pressure, 10 Hz pulse repetition frequency, 10% duty cycle, and a 6 s sonication time per spot. The focus of the transducer was 1.5 × 12 mm in the transverse and axial planes, respectively. Sham treatments were mice that received all injections and were placed under the ultrasound transducer, but no ultrasound was emitted.

**Figure 1:**
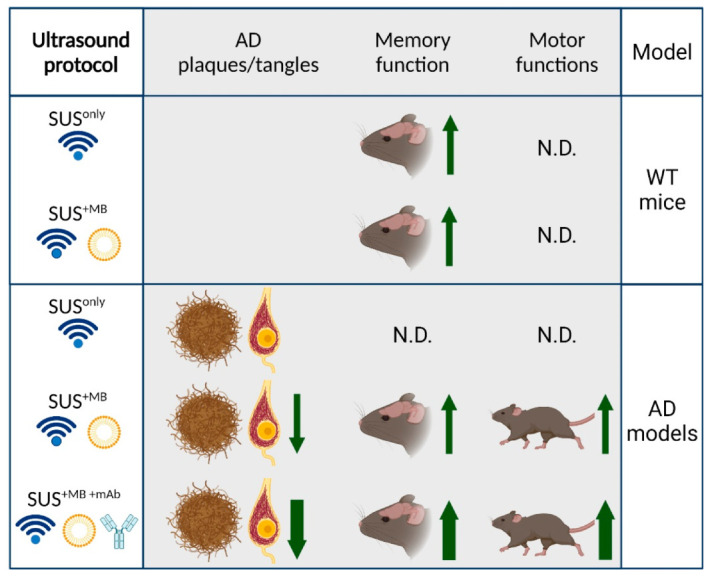
Three fundamental modes of ultrasound application (SUS^only^, SUS^+MB^, and SUS^+MB+mAb^) to wild-type mice and Alzheimer’s disease (AD) mouse models, showing the impact on AD pathology (plaques and tangles), memory, and motor functions. N.D.—not determined. Created with BioRender.com.

In our studies, we explored three fundamental modes of ultrasound application through the skull (Figure 1):

(a) SUS^only^: application of ultrasound only, which is a pressure wave that travels through the skull into the brain where the modality exerts bioeffects largely via radiation force.

(b) SUS^+MB^: application of ultrasound together with retro-orbitally or intravenously injected microbubbles (MB), which causes BBB opening of various degrees. Bioeffects are via cavitation (of microbubbles) but also via radiation force (as SUS^+MB^ as a modality inevitably includes SUS^only^). By opening the BBB, therapeutic (and other) blood-borne agents enter the brain and contribute to the observed bioeffects.

(c) SUS^+MB+mAb^: application of SUS+MB plus an intravenously injected therapeutic agent (e.g., an anti-Tau monoclonal antibody) whose brain uptake is facilitated in areas to which ultrasound is directed, allowing for local and global delivery.

We applied these three modes of ultrasound to AD mouse models and wild-type mice over a wide age range. The AD mouse models we used were (i) APP23 mice, which express a Swedish mutant form of APP and are characterized by amyloid plaque formation and memory impairment [56,57], and either (ii) pR5 mice, which express the P301L mutant form of Tau found in FTLD-Tau, a strain with tangle formation and memory impairment [58,59], or (iii) K3 mice, which express the K369I mutant form of Tau found in FTLD-Tau, a strain with tangle formation and memory impairment and aspects of Parkinsonism [60]. We used the C57Bl/6 strain as our wild-type (WT) mice.

**Figure 2:**
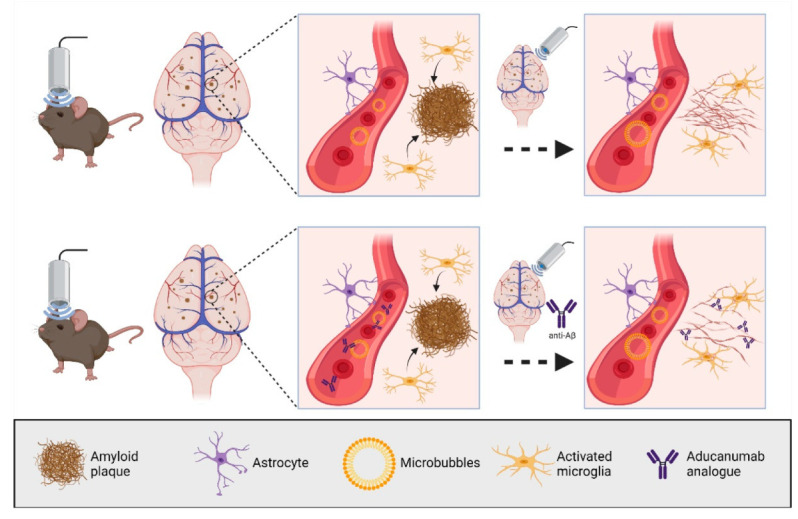
Impact of therapeutic ultrasound application on amyloid pathology. SUS^+MB^ facilitates the uptake of blood-borne factors, which activate microglia. These take up the amyloid into their lysosomes and digest it. Amyloid clearance and cognitive functions are improved when SUS^+MB^ is combined with an analogue of the anti-amyloid-β antibody aducanumab (SUS^+MB+ aducanumab analogue^). Created with BioRender.com.

**Figure 3:**
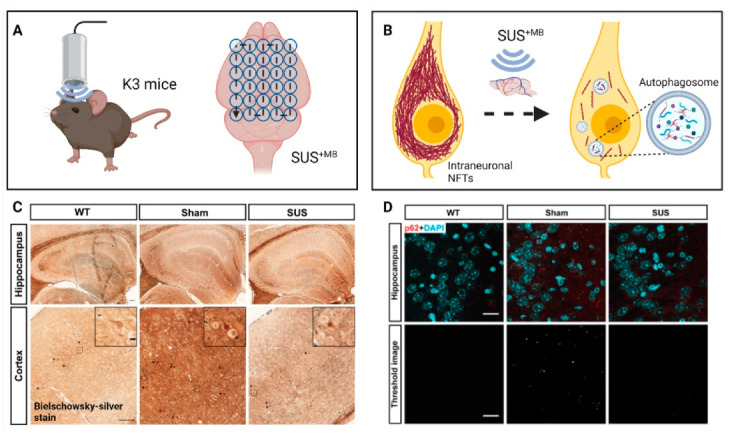
Impact of therapeutic ultrasound on Tau pathology. (**A**) The scanning ultrasound (SUS) path of the ultrasound beam is shown as the energy is applied to Tau transgenic K3 mice. (**B**) SUS with microbubbles (SUS^+MB^) clears Tau via the activation of neuronal autophagy. (**C**) Mice treated with SUS^+MB^ show reduced numbers of neurofibrillary tangle (NFT)-like inclusions detected by Bielschowsky-silver stain compared to sham controls. (**D**) The number and size of p62-positive puncta decrease upon SUS^+MB^ treatment compared to sham mice, indicative of induction of autophagy. Scale bar: 20 μm. (A) and (B) were created with BioRender.com. Figures (C) and (D) are adapted from [66], Ivyspring International Publisher, 2019.

Our studies in transgenic AD mouse models revealed that SUS+MB is a safe modality to clear both Aβ and Tau, albeit via the activation of different clearance mechanisms:

(a) In our first ultrasound study, we established the SUS paradigm (Figure 2). We showed that 5–8 weekly SUS^+MB^ treatments cleared Aβ in middle-aged APP23 mice via (as we assumed) unidentified blood-borne factors that entered the brain due to the transiently opened BBB, activating dormant brain microglia that took up the amyloid into their lysosomes and digested it. Plaque burden was reduced compared to sham-treated animals, and cleared plaques were observed in 75% of SUS^+MB^-treated mice. Because the histological and biochemical analysis was performed after several weekly treatments, microglial activation presents a snapshot at the time of analysis. We cannot formally rule out that additional clearance mechanisms were operating. Importantly, we found that spatial memory functions were restored in three complementary tests, novel object recognition, the Y-maze, and the active place avoidance (APA) test. Of note, there was no evidence of overt damage to the brain due to the sonication paradigm [4]. Similar approaches have been pursued by other groups [61,62]. The key message is that no exogenous agent was required to achieve the therapeutic benefit of SUS^+MB^.

(b) In an extension of this study, aged APP23 were used that are characterized by cerebral amyloid angiopathy (CAA), a hallmark lesion frequently observed in AD patients. Four weekly SUS^+MB^ sessions were found to clear Aβ, again by activating microglia. The treatment neither increased CAA (but also did not remove Aβ in the vessels) nor induced microbleeds. Memory functions had not been assessed in the study [63].

(c) Given that ultrasound without microbubbles is used as a neuromodulatory modality [64], we also explored SUS^only^ in APP23 mice, but found that this paradigm was not sufficient to clear amyloid. Whether or not SUS^only^ restores memory has not been addressed by us [65].

(d) A natural question arose whether SUS^+MB^ would also clear pathological Tau, which is deposited intraneuronally (Figure 3). By treating K3 mice 15 times weekly with SUS^+MB^, we found that Tau pathology was reduced without overt tissue damage. Associated impaired motor functions showed improvement towards the end of the treatment regime, with memory functions showing a trend towards improvement. In assessing potential clearance mechanisms, we ruled out a role for ubiquitination of Tau, a prerequisite for proteasomal clearance. However, the treatment regime induced the autophagy pathway in neurons as reflected by an increase in the autophagosome membrane marker LC3II and a reduction in the autophagic flux marker p62, along with a decrease of mTOR activity and an increase in beclin 1 levels. Moreover, there was a significant increase in the interaction of Tau and p62 in the ultrasound-treated mice, suggesting the removal of Tau by autophagosomes [66]. In an earlier study, we had treated pR5 mice with SUS^+MB^ as part of a study with four treatment arms. Again, tau was cleared compared to the sham control [67].

(e) We finally asked whether SUS^+MB^ would facilitate the uptake of anti-Tau antibodies and lead to increased therapeutic benefits (Figure 3). Indeed, such a paradigm, SUS^+MB + Tau mAbs^, challenged with different in-house anti-Tau antibodies in various antibody formats, revealed that therapeutic ultrasound significantly facilitates antibody uptake and reduces Tau pathology in K3 and pR5 mice [67,68,69]. Of note, as shown for the 2N Tau-specific antibody in a single chain variant (scFv) format, this approach not only increased brain uptake facilitated by therapeutic ultrasound but also uptake and distribution of a fluorescently labelled scFv into neuronal somata and neurites, as shown for the CA1 region of the hippocampus [67]. Brain uptake was massively increased with differences observed depending on the antibody format [68].

(f) Likewise, by cloning the aducanumab antibody, the SUS^+MB + aducanumab analogue^ paradigm revealed improved histopathological and behavioural outcomes for the combination therapy compared to the single treatment arms when the aducanumab analogue was delivered via ultrasound [70] (Figure 2). SUS^+MB + aducanumab analogue^ achieved at least a five-fold increased brain uptake of the antibody compared to the antibody-only arm. Of note, another group had also reported on an anti-Aβ antibody, which is not in a clinical trial [71].

Collectively, the data suggest that Aβ is an easier SUS target than Tau and that a combination therapy with therapeutic antibodies is more effective than the single treatment arms.

## 6. Therapeutic Improvements in Senescent Mice Using Therapeutic Ultrasound

Physiological ageing leads to a progressive decline in the functional and cellular constituents of the brain [19]. In a recent study, we explored the neuromodulatory potential of SUS in aged, cognitively impaired C57BL/6 wild-type mice [11]. Earlier studies in the lab in wild-type mice applied SUS^+MB^ and were intended to assess short- and long-term safety. They were conducted within a 4–9 month bracket [72] and within a 12–18 month bracket [73], respectively, using a range of technologies including electrophysiological recordings, Golgi stains, diffusion tensor imaging, magnetic resonance imaging (MRI), magnetic resonance spectroscopy, and spatial memory testing in the APA test. Collectively these studies indicated safety within the tested ultrasound parameter space.

With a maximal life-span of approximately 26 months [74], 12 month-old WT mice are still capable of spatial learning as revealed by the APA paradigm [73]. However, cognitive function subsequently deteriorates [75], and, by 18 months, spatial learning is severely compromised [76]. Similarly, LTP can be induced at 12 and 18 but not 20–22 months of age. We therefore used 20–22 month-old WT mice to determine whether therapeutic ultrasound can restore cognitive functions in the aged mouse brain. We made the remarkable observation that six weekly sessions of either SUS^+MB^ or, surprisingly, SUS^only^, restored LTP induction in these aged mice [11] (Figure 1). This demonstrates that in the physiologically aged brain, BBB opening, i.e., blood-borne factors entering the brain, is not required for the observed restorative effects of SUS. When spatial memory was assessed in the APA test, this showed a trend towards improved cognition in the SUS^+MB^ paradigm, whilst SUS^only^ resulted in statistically significant improvements. When a time-course of memory improvement was performed, this revealed gradual improvements as the number of after SUS^only^ treatment sessions was increased. Given that the study found changes to the NMDAR composition and proteomic changes conducive with LTP induction, and in light of a recent study by Oh and colleagues [77], the study suggests that ultrasound-mediated astrocytic glutamate release may signal through the NMDAR, which then leads to the observed therapeutic benefits. However, we cannot rule out additional mechanisms considering that we also found increased neurogenesis and alterations to the extracellular matrix. Collectively, our data suggest that low-intensity ultrasound restores LTP and memory in senescent mice through pleiotropic mechanisms including NMDAR signalling. This leads us to conclude that therapeutic ultrasound is a non-invasive, pleiotropic modality that may enhance cognition in the elderly. The study adds to the growing literature demonstrating that therapeutic ultrasound in the absence of microbubbles shows promise as a treatment for cognitive impairment [64].

## 7. Challenges and Opportunities in Developing Therapeutic Ultrasound into a Viable Therapy for Alzheimer’s Disease and Other Brain Diseases

In applying SUS^+MB^ to large animals (and by extension, human patients), one encounters several problems that need to be solved to develop the technique into a safe and effective modality. To achieve safety, such as the absence of bleeds, the variable skull attenuation needs to be accurately estimated to deliver acoustic energy within either a safe dosage window or a feedback control loop that adjusts the pressure as the ultrasound interacts with the circulating microbubbles. A second issue is the therapeutic window, which we believe for AD includes a large part of if not the entire human brain. The current technology does not allow quickly and effectively treating the entire brain, and some modalities have challenges in that they either target only superficial or deeply seated brain areas. A third challenge is in relation to the uniformity of BBB opening. There are many physiological factors that impact BBB opening that cannot be easily controlled such as local blood flow. Similarly, in the uptake of therapeutic agents, not only by the interstitial space but also by neurons, there is region- and cell-type-specific uptake, which needs to be controlled to include larger areas and more cell-types. Then, as discussed further up, not only are the toxic species of Aβ and Tau not fully defined, but some researchers would even dispute that these molecules have a role in AD at all. Accepting that they have a critical role in driving the disease process, a challenge is in clearing intraneuronal protein aggregates. Finally, there is a potential disconnect between aggregate clearance and the restoration of memory functions. Many current clinical studies using therapeutic ultrasound have no placebo (sham) control, and practice effects may suggest efficacy where in fact there may be none. These problems add to those generally encountered in trials of neurological diseases.

Over the last years, several groups have entered the clinical trial space. We are also taking steps towards achieving this goal, utilizing the insight we have gained into sheep as a large animal model given that the attenuating properties of a sheep skull to ultrasound are similar to those of a human skull [78]. Broadly speaking, there are three fundamental approaches in clinical trials: (i) an MRI-guided multi-array system as exemplified by Insightec’s ExaAblate Neuro device (NCT02986932, NCT03739905, NCT03671889), (ii) Carthera’s brain-implanted SonoCloud device (NCT03119961), and (iii) Columbia University’s neuronavigation-guided single-element FUS transducer. They all have their intrinsic advantages and limitations. The completed studies revealed the safety of delivering ultrasound with microbubbles, encouraging the entire field to move forward and refine the approach. Given the limited access to MRI scanners, patient compliance, and costs, developing a portable system would tick a lot of boxes. The challenge remains to safely and effectively treat large brain areas, if not the entire brain, within a reasonable amount of time. It remains to be determined how the ultrasound technology fairs in comparison with ongoing pharmacological and antibody strategies and whether the field will adopt combination therapies such as combining antibodies with therapeutic ultrasound. We remain optimistic that therapeutic ultrasound alone (administered as either SUS^only^ or SUS^+MB^), as well when co-administered with antibodies, holds great promise as an AD treatment. The work of our own lab and those cited in this review is a testament to the potential benefits therapeutic ultrasound may hold in conditions associated with ageing and more.

## Data Availability

Not applicable.

## References

[1] Götz J., Probst A., Spillantini M., Schäfer T., Jakes R., Bürki K., Goedert M. (1995). Somatodendritic localization and hyperphosphorylation of tau protein in transgenic mice expressing the longest human brain tau isoform. EMBO J..

[2] Chen X.-Q., Mobley W.C. (2019). Alzheimer Disease Pathogenesis: Insights from Molecular and Cellular Biology Studies of Oligomeric Aβ and Tau Species. Front. Neurosci..

[3] Polanco J.C., Li C., Bodea L.-G., Mármol R.M., Meunier F.A., Götz J. (2018). Amyloid-β and tau complexity—Towards improved biomarkers and targeted therapies. Nat. Rev. Neurol..

[4] Leinenga G., Götz J. (2015). Scanning ultrasound removes amyloid-β and restores memory in an Alzheimer’s disease mouse model. Sci. Transl. Med..

[5] O’Reilly M.A., Hynynen K. (2015). Emerging non-cancer applications of therapeutic ultrasound. Int. J. Hyperth..

[6] Lipsman N., Meng Y., Bethune A.J., Huang Y., Lam B., Masellis M., Herrmann N., Heyn C., Aubert I., Boutet A. (2018). Blood–brain barrier opening in Alzheimer’s disease using MR-guided focused ultrasound. Nat. Commun..

[7] Carpentier A., Canney M., Vignot A., Reina V., Beccaria K., Horodyckid C., Karachi C., Leclercq D., Lafon C., Chapelon J.-Y. (2016). Clinical trial of blood-brain barrier disruption by pulsed ultrasound. Sci. Transl. Med..

[8] Meng Y., Hynynen K., Lipsman N. (2021). Applications of focused ultrasound in the brain: From thermoablation to drug delivery. Nat. Rev. Neurol..

[9] Karakatsani M.E., Blesa J., Konofagou E.E. (2019). Blood–brain barrier opening with focused ultrasound in experimental models of Parkinson’s disease. Mov. Disord..

[10] Muñoz F., Aurup C., Konofagou E.E., Ferrera V.P. (2018). Modulation of Brain Function and Behavior by Focused Ultrasound. Curr. Behav. Neurosci. Rep..

[11] Blackmore D.G., Turpin F., Palliyaguru T., Evans H.T., Chicoteau A., Lee W., Pelekanos M., Nguyen N., Song J., Sullivan R.K.P. (2021). Low-intensity ultrasound restores long-term potentiation and memory in senescent mice through pleiotropic mechanisms including NMDAR signaling. Mol. Psychiatry.

[12] Feigin V.L., Vos T., Nichols E., Owolabi M.O., Carroll W.M., Dichgans M., Deuschl G., Parmar P., Brainin M., Murray C. (2020). The global burden of neurological disorders: Translating evidence into policy. Lancet Neurol..

[13] Goedert M. (2008). Oskar Fischer and the study of dementia. Brain.

[14] Arnsten A.F.T., Datta D., Del Tredici K., Braak H. (2021). Hypothesis: Tau pathology is an initiating factor in sporadic Alzheimer’s disease. Alzheimer’s Dement..

[15] Götz J., Bodea L.-G., Goedert M. (2018). Rodent models for Alzheimer disease. Nat. Rev. Neurosci..

[16] Sobue A., Komine O., Hara Y., Endo F., Mizoguchi H., Watanabe S., Murayama S., Saito T., Saido T.C., Sahara N. (2021). Microglial gene signature reveals loss of homeostatic microglia associated with neurodegeneration of Alzheimer’s disease. Acta Neuropathol. Commun..

[17] Saito T., Mihira N., Matsuba Y., Sasaguri H., Hashimoto S., Narasimhan S., Zhang B., Murayama S., Higuchi M., Lee V.M.Y. (2019). Humanization of the entire murine Mapt gene provides a murine model of pathological human tau propagation. J. Biol. Chem..

[18] Liu P.-P., Xie Y., Meng X.-Y., Kang J.-S. (2019). History and progress of hypotheses and clinical trials for Alzheimer’s disease. Signal Transduct. Target. Ther..

[19] López-Otín C., Blasco M.A., Partridge L., Serrano M., Kroemer G. (2013). The Hallmarks of Aging. Cell.

[20] Fjell A.M., Amlien I.K., Westlye L.T., Stenset V., Fladby T., Skinningsrud A., Eilsertsen D.E., Bjørnerud A., Walhovd K.B. (2010). CSF biomarker pathology correlates with a medial temporo-parietal network affected by very mild to moderate Alzheimer’s disease but not a fronto-striatal network affected by healthy aging. NeuroImage.

[21] Irwin K., Sexton C., Daniel T., Lawlor B., Naci L. (2018). Healthy Aging and Dementia: Two Roads Diverging in Midlife?. Front. Aging Neurosci..

[22] Ghosh K., Agarwal P., Haggerty G. (2011). Alzheimer’s Disease—Not an Exaggeration of Healthy Aging. Indian J. Psychol. Med..

[23] Cummings J., Lee G., Ritter A., Sabbagh M., Zhong K. (2020). Alzheimer’s disease drug development pipeline: 2020. Alzheimer’s Dement. Transl. Res. Clin. Interv..

[24] Arndt J.W., Qian F., Smith B.A., Quan C., Kilambi K.P., Bush M.W., Walz T., Pepinsky R.B., Bussière T., Hamann S. (2018). Structural and kinetic basis for the selectivity of aducanumab for aggregated forms of amyloid-β. Sci. Rep..

[25] Sevigny J., Chiao P., Bussière T., Weinreb P.H., Williams L., Maier M., Dunstan R., Salloway S., Chen T., Ling Y. (2016). The antibody aducanumab reduces Aβ plaques in Alzheimer’s disease. Nature.

[26] Linse S., Scheidt T., Bernfur K., Vendruscolo M., Dobson C.M., Cohen S.I.A., Sileikis E., Lundqvist M., Qian F., O’Malley T. (2020). Kinetic fingerprints differentiate the mechanisms of action of anti-Aβ antibodies. Nat. Struct. Mol. Biol..

[27] Schneider L. (2020). A resurrection of aducanumab for Alzheimer’s disease. Lancet Neurol..

[28] Howard R., Liu K.Y. (2019). Questions EMERGE as Biogen claims aducanumab turnaround. Nat. Rev. Neurol..

[29] Holmes C., Boche D., Wilkinson D., Yadegarfar G., Hopkins V., Bayer A., Jones R.W., Bullock R., Love S., Neal J.W. (2008). Long-term effects of Aβ42 immunisation in Alzheimer’s disease: Follow-up of a randomised, placebo-controlled phase I trial. Lancet.

[30] Hanseeuw B.J., Betensky R.A., Jacobs H.I.L., Schultz A.P., Sepulcre J., Becker J.A., Cosio D.M.O., Farrell M., Quiroz Y.T., Mormino E.C. (2019). Association of Amyloid and Tau with Cognition in Preclinical Alzheimer Disease: A Longitudinal Study. JAMA Neurol..

[31] Weisová P., Cehlár O., Škrabana R., Zilkova M., Filipčík P., Kováčech B., Prčina M., Wojčiaková L., Fialová L., Smolek T. (2019). Therapeutic antibody targeting microtubule-binding domain prevents neuronal internalization of extracellular tau via masking neuron surface proteoglycans. Acta Neuropathol. Commun..

[32] Yanamandra K., Kfoury N., Jiang H., Mahan T., Ma S., Maloney P.S.E., Wozniak D.F., Diamond M.I., Holtzman D.M. (2013). Anti-Tau Antibodies that Block Tau Aggregate Seeding In Vitro Markedly Decrease Pathology and Improve Cognition In Vivo. Neuron.

[33] Courade J.-P., Angers R., Mairet-Coello G., Pacico N., Tyson K., Lightwood D., Munro R., McMillan D., Griffin R., Baker T. (2018). Epitope determines efficacy of therapeutic anti-Tau antibodies in a functional assay with human Alzheimer Tau. Acta Neuropathol..

[34] Höglinger G.U., Litvan I., Mendonca N., Wang D., Zheng H., Rendenbach-Mueller B., Lon H.-K., Jin Z., Fisseha N., Budur K. (2021). Safety and efficacy of tilavonemab in progressive supranuclear palsy: A phase 2, randomised, placebo-controlled trial. Lancet Neurol..

[35] Kim B., Mikytuck B., Suh E., Gibbons G.S., Van Deerlin V.M., Vaishnavi S.N., Spindler M.A., Massimo L., Grossman M., Trojanowski J.Q. (2021). Tau immunotherapy is associated with glial responses in FTLD-tau. Acta Neuropathol..

[36] Cope T.E., Rittman T., Borchert R.J., Jones P.S., Vatansever D., Allinson K., Passamonti L., Rodriguez P.V., Bevan-Jones W.R., O’Brien J. (2018). Tau burden and the functional connectome in Alzheimer’s disease and progressive supranuclear palsy. Brain.

[37] Barthélemy N.R., Gabelle A., Hirtz C., Fenaille F., Sergeant N., Schraen-Maschke S., Vialaret J., Buee L., Junot C., Becher F. (2016). Differential Mass Spectrometry Profiles of Tau Protein in the Cerebrospinal Fluid of Patients with Alzheimer’s Disease, Progressive Supranuclear Palsy, and Dementia with Lewy Bodies. J. Alzheimer’s Dis..

[38] Pascual G., Wadia J.S., Zhu X., Keogh E., Kükrer B., Van Ameijde J., Inganäs H., Siregar B., Perdok G., Diefenbach O. (2017). Immunological memory to hyperphosphorylated tau in asymptomatic individuals. Acta Neuropathol..

[39] Pandit R., Chen L., Götz J. (2020). The blood-brain barrier: Physiology and strategies for drug delivery. Adv. Drug Deliv. Rev..

[40] Kaur C., Rathnasamy G., Ling E.-A. (2016). The Choroid Plexus in Healthy and Diseased Brain. J. Neuropathol. Exp. Neurol..

[41] Lun M.P., Monuki E.S., Lehtinen M.K. (2015). Development and functions of the choroid plexus–cerebrospinal fluid system. Nat. Rev. Neurosci..

[42] Golde T.E. (2014). Open questions for Alzheimer’s disease immunotherapy. Alzheimer’s Res. Ther..

[43] Dewhirst M.W., Viglianti B.L., Lora-Michiels M., Hanson M., Hoopes P.J. (2003). Basic principles of thermal dosimetry and thermal thresholds for tissue damage from hyperthermia. Int. J. Hyperth..

[44] Elias W.J., Huss D., Voss T., Loomba J., Khaled M., Zadicario E., Frysinger R.C., Sperling S., Wylie S., Monteith S.J. (2013). A Pilot Study of Focused Ultrasound Thalamotomy for Essential Tremor. N. Engl. J. Med..

[45] Elias W.J., Lipsman N., Ondo W.G., Ghanouni P., Kim Y.G., Lee W., Schwartz M., Hynynen K., Lozano A., Shah B.B. (2016). A Randomized Trial of Focused Ultrasound Thalamotomy for Essential Tremor. N. Engl. J. Med..

[46] Chang K.W., Jung H.H., Chang J.W. (2021). Magnetic Resonance-Guided Focused Ultrasound Surgery for Obsessive-Compulsive Disorders: Potential for use as a Novel Ablative Surgical Technique. Front. Psychiatry.

[47] Yulug B., Hanoglu L., Kilic E. (2017). The neuroprotective effect of focused ultrasound: New perspectives on an old tool. Brain Res. Bull..

[48] Bakay L., Hueter T.F., Ballantine H.T., Sosa D. (1956). Ultrasonically Produced Changes in the Blood-Brain Barrier. Arch. Neurol. Psychiatry.

[49] Hynynen K., McDannold N., Vykhodtseva N., Jolesz F.A. (2001). Noninvasive MR Imaging–guided Focal Opening of the Blood-Brain Barrier in Rabbits. Radiology.

[50] Sheikov N., McDannold N., Vykhodtseva N., Jolesz F., Hynynen K. (2004). Cellular mechanisms of the blood-brain barrier opening induced by ultrasound in presence of microbubbles. Ultrasound Med. Biol..

[51] Deng J., Huang Q., Wang F., Liu Y., Wang Z., Wang Z., Zhang Q., Lei B., Cheng Y. (2011). The Role of Caveolin-1 in Blood–Brain Barrier Disruption Induced by Focused Ultrasound Combined with Microbubbles. J. Mol. Neurosci..

[52] Pandit R., Koh W.K., Sullivan R.K., Palliyaguru T., Parton R.G., Götz J. (2020). Role for caveolin-mediated transcytosis in facilitating transport of large cargoes into the brain via ultrasound. J. Control. Release.

[53] Fry W.J., Mosberg W.H., Barnard J.W., Fry F.J. (1954). Production of Focal Destructive Lesions in the Central Nervous System with Ultrasound. J. Neurosurg..

[54] Leinenga G., Langton C., Nisbet R., Götz G.L.R.N.J. (2016). Ultrasound treatment of neurological diseases—current and emerging applications. Nat. Rev. Neurol..

[55] Seip R., Chin C.T., Hall C., Raju B., Ghanem A., Tiemann K. (2009). Targeted Ultrasound-Mediated Delivery of Nanoparticles: On the Development of a New HIFU-Based Therapy and Imaging Device. IEEE Trans. Biomed. Eng..

[56] Sturchler-Pierrat C., Abramowski D., Duke M., Wiederhold K.-H., Mistl C., Rothacher S., Ledermann B., Bürki K., Frey P., Paganetti P. (1997). Two amyloid precursor protein transgenic mouse models with Alzheimer disease-like pathology. Proc. Natl. Acad. Sci. USA.

[57] Ittner L.M., Ke Y., Delerue F., Bi M., Gladbach A., van Eersel J., Wölfing H., Chieng B.C., Christie M., Napier I.A. (2010). Dendritic Function of Tau Mediates Amyloid-β Toxicity in Alzheimer’s Disease Mouse Models. Cell.

[58] Pennanen L., Welzl H., D’Adamo P., Nitsch R.M., Götz J. (2004). Accelerated extinction of conditioned taste aversion in P301L tau transgenic mice. Neurobiol. Dis..

[59] Deters N., Ittner L.M., Götz J., Gtz J. (2008). Divergent phosphorylation pattern of tau in P301L tau transgenic mice. Eur. J. Neurosci..

[60] Ittner L.M., Ke Y., Götz J. (2009). Phosphorylated Tau Interacts with c-Jun N-terminal Kinase-interacting Protein 1 (JIP1) in Alzheimer Disease. J. Biol. Chem..

[61] Jordão J.F., Thévenot E., Markham-Coultes K., Scarcelli T., Weng Y.-Q., Xhima K., O’Reilly M., Huang Y., McLaurin J., Hynynen K. (2013). Amyloid-β plaque reduction, endogenous antibody delivery and glial activation by brain-targeted, transcranial focused ultrasound. Exp. Neurol..

[62] Burgess A., Dubey S., Yeung S., Hough O., Eterman N., Aubert I., Hynynen K. (2014). Alzheimer Disease in a Mouse Model: MR Imaging–guided Focused Ultrasound Targeted to the Hippocampus Opens the Blood-Brain Barrier and Improves Pathologic Abnormalities and Behavior. Radiology.

[63] Leinenga G., Götz J. (2018). Safety and Efficacy of Scanning Ultrasound Treatment of Aged APP23 Mice. Front. Neurosci..

[64] Tyler W.J., Lani S.W., Hwang G.M. (2018). Ultrasonic modulation of neural circuit activity. Curr. Opin. Neurobiol..

[65] Leinenga G., Koh W.K., Götz J. (2019). Scanning ultrasound in the absence of blood-brain barrier opening is not sufficient to clear β-amyloid plaques in the APP23 mouse model of Alzheimer’s disease. Brain Res. Bull..

[66] Pandit R., Leinenga G., Götz J. (2019). Repeated ultrasound treatment of tau transgenic mice clears neuronal tau by autophagy and improves behavioral functions. Theranostics.

[67] Nisbet R.M., Van der Jeugd A., Leinenga G., Evans H., Janowicz P., Götz J. (2017). Combined effects of scanning ultrasound and a tau-specific single chain antibody in a tau transgenic mouse model. Brain.

[68] Janowicz P.W., Leinenga G., Götz J., Nisbet R.M. (2019). Ultrasound-mediated blood-brain barrier opening enhances delivery of therapeutically relevant formats of a tauspecific antibody. Sci. Rep..

[69] Bajracharya R., Brici D., Bodea L.-G., Janowicz P.W., Götz J., Nisbet R.M. (2021). Tau antibody isotype induces differential effects following passive immunisation of tau transgenic mice. Acta Neuropathol. Commun..

[70] Leinenga G., Koh W.K., Götz J. (2021). A comparative study of the effects of Aducanumab and scanning ultrasound on amyloid plaques and behavior in the APP23 mouse model of Alzheimer disease. Alzheimer’s Res. Ther..

[71] Jordão J.F., Ayala-Grosso C.A., Markham K., Huang Y., Chopra R., McLaurin J., Hynynen K., Aubert I. (2010). Antibodies Targeted to the Brain with Image-Guided Focused Ultrasound Reduces Amyloid-β Plaque Load in the TgCRND8 Mouse Model of Alzheimer’s Disease. PLoS ONE.

[72] Hatch R.J., Leinenga G., Götz J. (2016). Scanning Ultrasound (SUS) Causes No Changes to Neuronal Excitability and Prevents Age-Related Reductions in Hippocampal CA1 Dendritic Structure in Wild-Type Mice. PLoS ONE.

[73] Blackmore D.G., Turpin F., Mohamed A.Z., Zong F., Pandit R., Pelekanos M., Nasrallah F., Sah P., Bartlett P.F., Götz J. (2018). Multimodal analysis of aged wild-type mice exposed to repeated scanning ultrasound treatments demonstrates long-term safety. Theranostics.

[74] Turturro A., Duffy P., Hass B., Kodell R., Hart R. (2002). Survival characteristics and age-adjusted disease incidences in C57BL/6 mice fed a commonly used cereal-based diet modulated by dietary restriction. J. Gerontol. Ser. A Boil. Sci. Med. Sci..

[75] Benice T., Rizk A., Kohama S., Pfankuch T., Raber J. (2006). Sex-differences in age-related cognitive decline in C57BL/6J mice associated with increased brain microtubule-associated protein 2 and synaptophysin immunoreactivity. Neuroscience.

[76] Van Praag H., Shubert T.E., Zhao C., Gage F.H. (2005). Exercise Enhances Learning and Hippocampal Neurogenesis in Aged Mice. J. Neurosci..

[77] Oh S.-J., Lee J.M., Kim H.-B., Lee J., Han S., Bae J.Y., Hong G.-S., Koh W., Kwon J., Hwang E.-S. (2019). Ultrasonic Neuromodulation via Astrocytic TRPA1. Curr. Biol..

[78] Pelekanos M., Leinenga G., Odabaee M., Odabaee M., Saifzadeh S., Steck R., Götz J. (2018). Establishing sheep as an experimental species to validate ultrasound-mediated blood-brain barrier opening for potential therapeutic interventions. Theranostics.

